# Cooperative interaction of BMP signalling and *Foxn1* gene dosage determines the size of the functionally active thymic epithelial compartment

**DOI:** 10.1038/s41598-017-09213-1

**Published:** 2017-08-17

**Authors:** Jeremy B. Swann, Brigitte Krauth, Christiane Happe, Thomas Boehm

**Affiliations:** Department of Developmental Immunology, Max Planck Institute of Immunobiology and Epigenetics, Stuebeweg 51, D-79108 Freiburg, Germany

## Abstract

Thymopoiesis strictly depends on the function of the Foxn1 transcription factor that is expressed in the thymic epithelium. During embryonic development, initial expression of the *Foxn1* gene is induced in the pharyngeal endoderm by mesenchyme-derived BMP4 signals. Here, by engineering a time-delayed feedback system of BMP inhibition in mouse embryos, we demonstrate that thymopoiesis irreversibly fails if *Foxn1* gene expression does not occur during a defining time span in mid-gestation. We also reveal an epistatic interaction between the extent of BMP signalling and the gene dosage of *Foxn1*. Our findings illustrate the complexities of the early steps of thymopoiesis and indicate that sporadic forms of thymic hypoplasia in humans may result from the interaction of genes affecting the magnitude of BMP signalling and *Foxn1* expression.

## Introduction

In all vertebrates, the thymus is the site of T cell development^1^. The epithelial thymic rudiment arises from the endoderm of the third pharyngeal pouch, and, under the influence of the Foxn1 transcription factor, eventually differentiates into functionally active thymic epithelial cells^2–4^. The thymic microenvironment attracts lymphoid precursor cells, specifies them to the T cell lineage, and orchestrates a complex series of selection events that culminates in the generation of self-tolerant T cells, collectively expressing a clonally dispersed and structurally diverse repertoire of T cell antigen receptors^5^.

Early in embryonic development, the primordial endoderm emits signals (possibly including Fgf8) to the adjacent mesenchyme, which leads to the induction of BMP4 expression^6^. Subsequently, BMP4 signals back to the endodermal compartment to initiate the expression of *Foxn1*, the gene encoding a key transcriptional regulator required for the differentiation of the future thymic epithelium^2–4^. The mesenchymal contribution to thymopoiesis is crucial, as demonstrated by the formation of poorly developed thymic lobes after ablation of the neural crest in chicken^7^, dysplasia of the thymus after inhibition of BMP signalling in the pharyngeal arches^8^ or in the thymic rudiment^9, 10^, failure of T cell differentiation in cultured fetal thymic lobes after removal of the mesenchyme^11^, and dysplasia of the thymus in the chromosome 22 deletion (also known as DiGeorge) syndrome^12^. Interestingly, however, the requirement for BMP4 signalling from the mesenchyme appears to be only transient, at least in chicken^6^. The same may hold true in mice, since *Foxn1* expression is maintained after deletion of *Bmp4* in the mesenchymal compartment through the use of a *Foxg1:Cre* transgene^13^, although this depletion may only be partial. Collectively, these data indicate that after a short inductive period, *Foxn1* expression becomes independent of BMP4.

Here, we set out to answer some of the unresolved questions of early thymus development. For instance, it is not known whether the function of Foxn1 itself is required during the sensitive phase of induction, or whether other factor(s) induced by BMP4 signalling are required to establish thymic epithelial fate. Moreover, it is unclear whether the activity of the dysplastic thymus resulting from perturbed BMP4 signalling during embryogenesis recovers during later stages of development, for instance by BMP4 signals arising from tissue adjacent to the thymus. We have addressed these questions by examining the possible presence of epistasis between BMP4 signals and *Foxn1* gene dosage, and its functional consequences in adult life. Our results indicate that failure of stable *Foxn1* expression irreversibly converts the prospective thymic anlage into a lymphopoietically-deficient organ rudiment.

## Results

### Specification of thymic epithelial cells by BMP4 signalling

**Figure 1:**
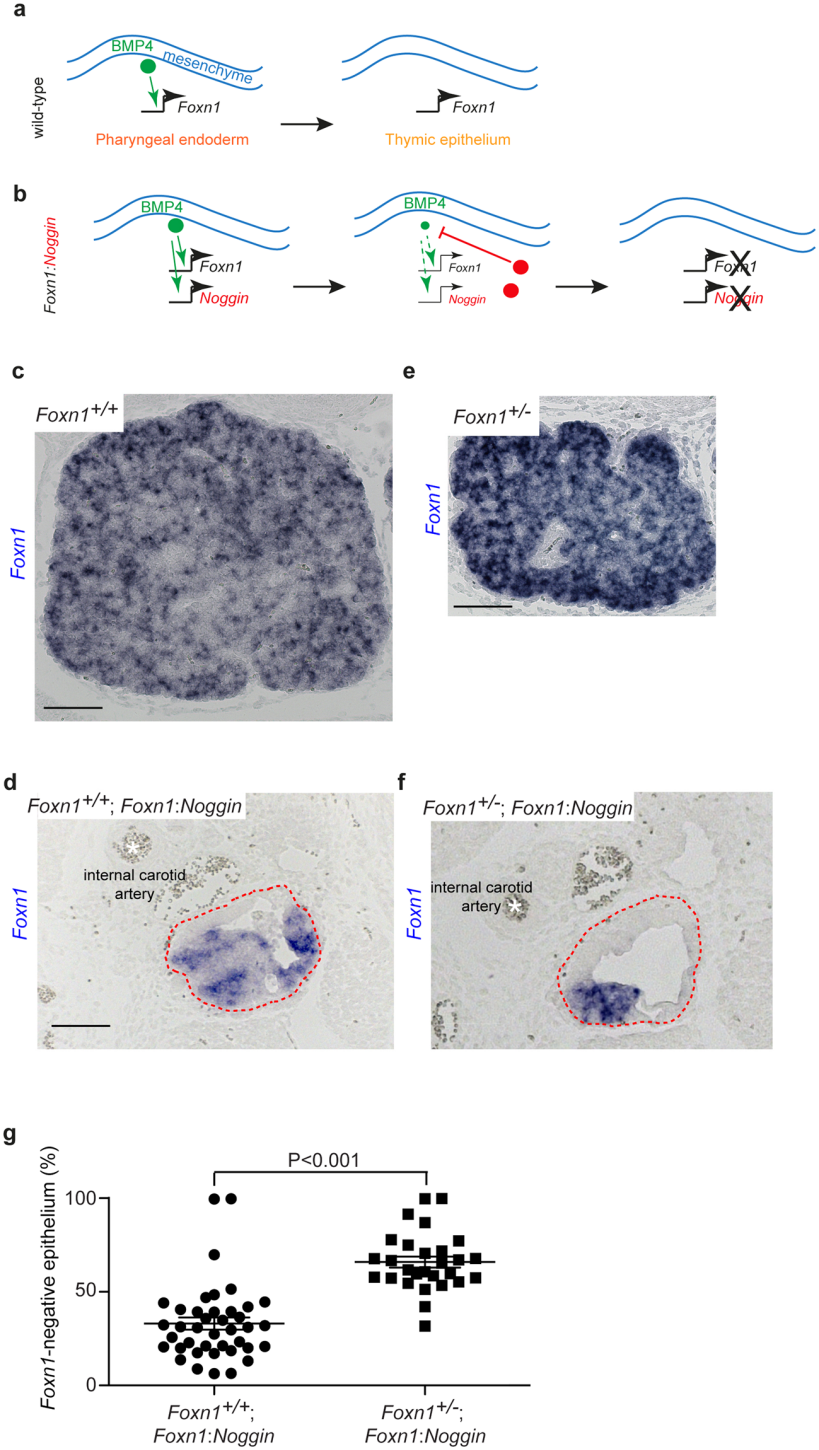
Role of BMP signalling and *Foxn1* dosage in thymus development. (**a**) Schematic of the developmental sequence giving rise to the initiation of *Foxn1* expression in the future thymic epithelium (for details, see text). (**b**) Schematic illustrating the time-delayed feedback inhibition system enabled by the transgenic expression of the BMP inhibitor NOGGIN under the transcriptional control of the *Foxn1* gene promoter (for details, see text). Panels (c) to (f) depict results of RNA *in situ* hybridization using a *Foxn1*-specific probe (positive cells stained blue). (**c**) At day 15.5 of embryonic development (E15.5), the thymic epithelium of wild-type (*Foxn1* ^+/+^) mice uniformly expresses *Foxn1*. (**d**) Generation of *Foxn1*-negative thymic epithelia through time-delayed feedback inhibition in the thymic anlage (encircled with dotted red lines) at E15.5, as revealed by RNA *in situ* hybridization in *Foxn1* ^+/+^;*Foxn1:Noggin* mice. (**e**) At E15.5, the thymic epithelium of *Foxn1*-heterozygous (*Foxn1* ^+/−^) mice uniformly expresses *Foxn1*, although the rudiment tends to be smaller^16–20^ than in *Foxn1* ^+/+^ siblings (see Fig. 2). (**f**) In E15.5 *Foxn1* ^+/−^;*Foxn1:Noggin* transgenics, the *Foxn1*-negative domain is larger than in *Foxn1* ^+/+^;*Foxn1:Noggin* transgenics. For orientation, the position of the internal carotid artery is indicated with a white asterix in panels (d) and (f), and the thymic anlagen are encircled with dashed red lines. (**g**) *Foxn1* heterozygosity in *Foxn1:Noggin* transgenics increases the *Foxn1*-negative fraction of the thymic epithelium from 33.1 ± 3.2% (mean± s.e.m.) to 65.9 ± 2.9% (mean ± s.e.m.); see Supplementary Figs 3 and 4 for original data underlying these summary statistics. Scale bars, 100 μm.

Thymic epithelial cells (TECs) are characterized by the expression of the gene encoding the transcription factor Foxn1. During embryonic development, BMP4 emanating from the mesenchyme of the pharyngeal pouch induces the expression of *Foxn1* in the underlying endoderm to establish the future thymic microenvironment^6^ (Fig. 1a). At embryonic day 13.5 of development (E13.5), BMP4 expression is detectable in the epithelial thymic rudiment and its mesenchymal capsule, although the expression levels in individual cells of the epithelium are variable^9^ (Supplementary Fig. 1a); by contrast, BMP4 is not expressed in the adjacent anlage of the parathyroid^14^. In the present context, it is important to point out that BMP4 expression is also readily detectable in the non-functional thymic anlage of *Foxn1* ^−/−^ mice (Supplementary Fig. 1b,c), supporting the notion that BMP4 functions upstream of *Foxn1* (Fig. 1a).

In order to dampen the initial period of BMP4 signalling during early thymus development, we expressed NOGGIN, a known inhibitor of BMP4, in thymic epithelial cells under the control of the *Foxn1* promoter (Fig. 1b). In this situation, initial BMP signalling from the mesenchyme activates not only the endogenous *Foxn1* gene, but also the *Foxn1:Noggin* transgene in the future thymic epithelium (Fig. 1b). Hence, this creates a time-delayed feedback inhibition of BMP signalling via the production of NOGGIN in epithelial cells. In contrast to the situation in wild-type mice, in which all TECs are *Foxn1*-positive at this stage of development^4, 15^ (Fig. 1c), the condition of reduced BMP4 signalling has transformed the thymic rudiment into a mosaic comprising *Foxn1*-positive and *Foxn1*-negative epithelial cells^9^ (Fig. 1d); of note, it was previously shown that the *Foxn1*-negative cells in the cystic rudiment arise as a consequence of the failure to maintain *Foxn1* expression^10^. Hence, sufficient levels of BMP4 signalling are required during early development to establish an epithelial domain stably expressing *Foxn1*. Because the levels of *Bmp4* expression appear to vary in different parts of the embryonic thymic anlage, the extent of suppression of BMP4 signalling (resulting from different degrees of competition with the inhibitor) likewise varies; cells with the highest levels of *Bmp4* expression in the wild-type embryo^9^ exhibit the greatest resistance to the inhibitory effects of NOGGIN.

The secreted BMP4 inhibitor NOGGIN does not perturb the function of the parathyroid despite the fact that the parathyroid is - like the thymus - a derivative of the third pharyngeal pouch endoderm and develops in close apposition to the thymic anlage; it is conceivable that in the transgenic situation increased levels of the BMP inhibitor are functionally irrelevant for the specification of the parathyroid (which is revealed by the activity of the *Gcm2* transcription factor gene^14^), because the epithelium of the parathyroid itself expresses the Noggin gene^14^. Reduced BMP activity in the thymic domain, however, affects the separation of the thymic and parathyroid rudiments; while most *Gcm2*-expressing cells form a compact organ rudiment, some *Gcm2*-positive cells can be found in or near the *Foxn1*-expressing region (Supplementary Fig. 2a). However, this extrathymic effect of BMP inhibition is not related to the emergence of *Foxn1*-deficient epithelium in the transgenic thymic anlage, as the separation of the thymus and parathyroid occurs normally in *Foxn1* ^−/−^ mice (Supplementary Fig. 2b,c), and hence is due to a function genetically upstream of *Foxn1*.

### BMP signalling and Foxn1 dosage cooperate to establish the size of the TEC progenitor compartment

The incomplete loss of *Foxn1* expression in the thymic anlage under conditions of limiting BMP signalling (Fig. 1b) afforded us the possibility to directly examine the role of FOXN1 dosage in the emergence of epithelial cells stably expressing *Foxn1*. To this end, we crossed the *Foxn1:Noggin* transgene onto the *Foxn1* ^+/−^ background, in which only one allele of the *Foxn1* gene is active^4^, and accompanied by a mild reduction of thymus size, the extent of which depends on species and strain^16–20^. Note, however, that this reduction is not caused by the appearance of *Foxn1*-negative epithelium in *Foxn1* heterozygous mice, since the density of *Foxn1*-expressing TECs in the thymic rudiment is indistinguishable from that of their *Foxn1* ^+/+^ counterpart (c.f., Fig. 1c and Fig. 1e). By contrast, the thymic anlage of heterozygous *Foxn1* ^+/−^; *Foxn1:Noggin* transgenic mice contains twice as many *Foxn1*-negative TECs (Fig. 1f) in comparison to their *Foxn1* ^+/+^; *Foxn1:Noggin* siblings (Fig. 1g; Supplementary Figs 3 and 4), suggesting that a larger fraction of TECs fail to maintain *Foxn1* expression under the condition of reduced *Foxn1* dosage. Collectively, these results indicate that the extent of BMP signalling and *Foxn1* gene dosage cooperate in early embryogenesis to establish the number of epithelial cells stably expressing *Foxn1*, the hallmark of a functionally competent thymic epithelium^4^.

### Impaired BMP signalling during embryogenesis permanently reduces the number of thymopoietically active epithelial cells

Collectively, the above results indicate that under the condition of limiting BMP4 signalling in *Foxn1:Noggin* transgenic thymi, fewer TEC progenitors are established in early embryogenesis, a situation quantitatively equivalent to that resulting from sub-total TEC ablation^15^. It was of particular interest, therefore, to examine whether the small numbers of TEC progenitors would be capable of expanding after the BMP-sensitive period of development^6^ to eventually restore near-normal numbers of thymopoietically active TECs.

**Figure 2:**
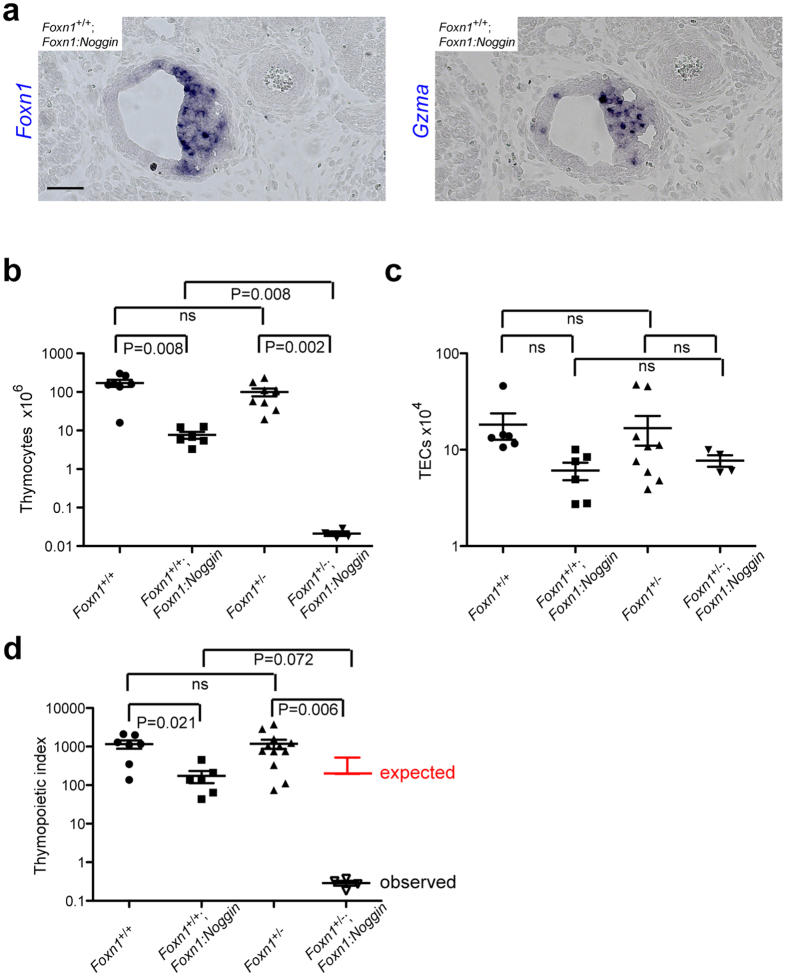
Thymopoietic activity in the rudiment of *Foxn1:Noggin* transgenic mice. (**a**) RNA *in situ* hybridization of the thymic rudiments of *Foxn1* ^+/+^; *Foxn1:Noggin* transgenic embryos at E15.5; positive cells are stained blue. The probes used are indicated to the left; genotypes are indicated in the top left corner. Scale bar, 100 μm. (**b**) Number of CD45^+^ thymocytes. Intrathymic T cell development is qualitatively normal in transgenic mice (Supplementary Fig. 5). (**c**) Number of thymic epithelial cells (TECs). (**d**) Thymopoietic indices, calculated as thymocyte/TEC ratios. The mean + s.e.m. value expected under the multiplicative model of epistasis^22^ is indicated in red; the difference to the observed values is estimated to be significant at P < 0.01. In (**b**) to (**d**), means ± s.e.m for 4-week-old mice.

*Foxn1*-deficient TECs do not possess thymopoietic activity^4, 15^; hence, T cell development in *Foxn1:Noggin* transgenic thymi is supported only by the small number of *Foxn1*-positive TECs remaining in the rudiment, as revealed by the expression of the early thymocyte marker *granzyme A* ^21^ (Fig. 2a). Owing to the difficulties encountered in reliably determining the numbers of haematopoietic cells and TECs in the ectopic thymic lobes of *Foxn1:Noggin* transgenic embryos, we quantified thymopoietic activity in transgenic mice at 4 weeks of age. At this stage, the thymus of *Foxn1* ^+/+^ wild-type mice contains approximately 1.2 × 10^8^ thymocytes, whereas only approximately 9 × 10^6^ thymocytes can be recovered from the thymus of *Foxn1* ^+/+^;*Foxn1:Noggin* transgenics (Fig. 2b); note that in our previous study^9^, the precise numbers of thymocytes in the ectopic thymi were not recorded. Interestingly, under conditions of *Foxn1* heterozygosity, the deleterious effect of the *Foxn1:Noggin* transgene on thymocyte numbers is more severe; the cellularities of *Foxn1* ^+/−^ and *Foxn1* ^+/−^;*Foxn1:Noggin* thymi differ by more than 3 orders of magnitude, resulting in less than 10^4^ thymocytes per rudiment (Fig. 2b). Because the numbers of TECs in the thymi of the four genotypes vary only slightly (the differences did not achieve statistical significance; Fig. 2c), the considerably lower thymopoietic indices (number of thymocytes per TEC) likely reflect the presence of a large proportion of functionally inactive TECs in the *Foxn1:Noggin* transgenics (Fig. 2d). Although *Foxn1*-negative TECs are already apparent during embryonic development (Fig. 1; Supplementary Figs 3 and 4), the discrepancies between the thymopoietic capacities of *Foxn1*-sufficient and *Foxn1* heterozygous transgenics is striking. Numerically, whereas one TEC in the wild-type situation supports on average about 1,000 thymocytes, one TEC in *Foxn1* ^+/−^;*Foxn1:Noggin* thymi supports less than 1 thymocyte. Hence, assuming that the thymopoietic capacities of *Foxn1*-expressing TECs that remain in the transgenic thymi are similar to those of their non-transgenic counterparts, we estimate that at 4 weeks of age approximately 15% of TECs remain functional (i.e., stably express *Foxn1*) in the *Foxn1* ^+/+^;*Foxn1:Noggin* genotype, whereas in the *Foxn1* ^+/−^;*Foxn1:Noggin* genotype, the same is true for less than 0.1% of TECs. Nonetheless, despite their reduced numbers, thymocytes execute their normal developmental programme in the transgenic environments (Supplementary Fig. 5).

### Epistatic interaction of BMP signalling and Foxn1 copy number

Genetic interaction analysis can be used to infer the mechanism by which two or more perturbations affect a particular phenotype^22^. In genetic interaction analysis, a so-called synthetic interaction is said to occur when a double mutant exhibits a more extreme phenotype than expected from single-mutant phenotypes; in this case, the two genes often function in distinct but compensatory pathways. By contrast, in the case of alleviating or suppressing interactions, the phenotype is less severe than would have been expected from the individual contributions. The latter types of interactions often occur when the gene products function in the same pathway, so that the mutation in one gene masks or suppresses the effects of the mutation in the other. The thymopoietic index of *Foxn1* ^+/−^;*Foxn1:Noggin* mice is reduced more than 1,000 fold (Fig. 2d) compared to that of wild-type mice; this reduction is much greater than would have been expected from the effects of the combined individual contributions, that is, *Foxn1* heterozygosity (no change) and the *Foxn1:Noggin* transgene (less than 10 fold reduction). Considering the observed synthetic interaction in the context of prevailing theories of genetic interactions^22^, we conclude that BMP signalling and *Foxn1* dosage predominantly act in parallel pathways converging on the number of functional TECs.

### Loss of lineage identity in thymic epithelial cells

**Figure 3:**
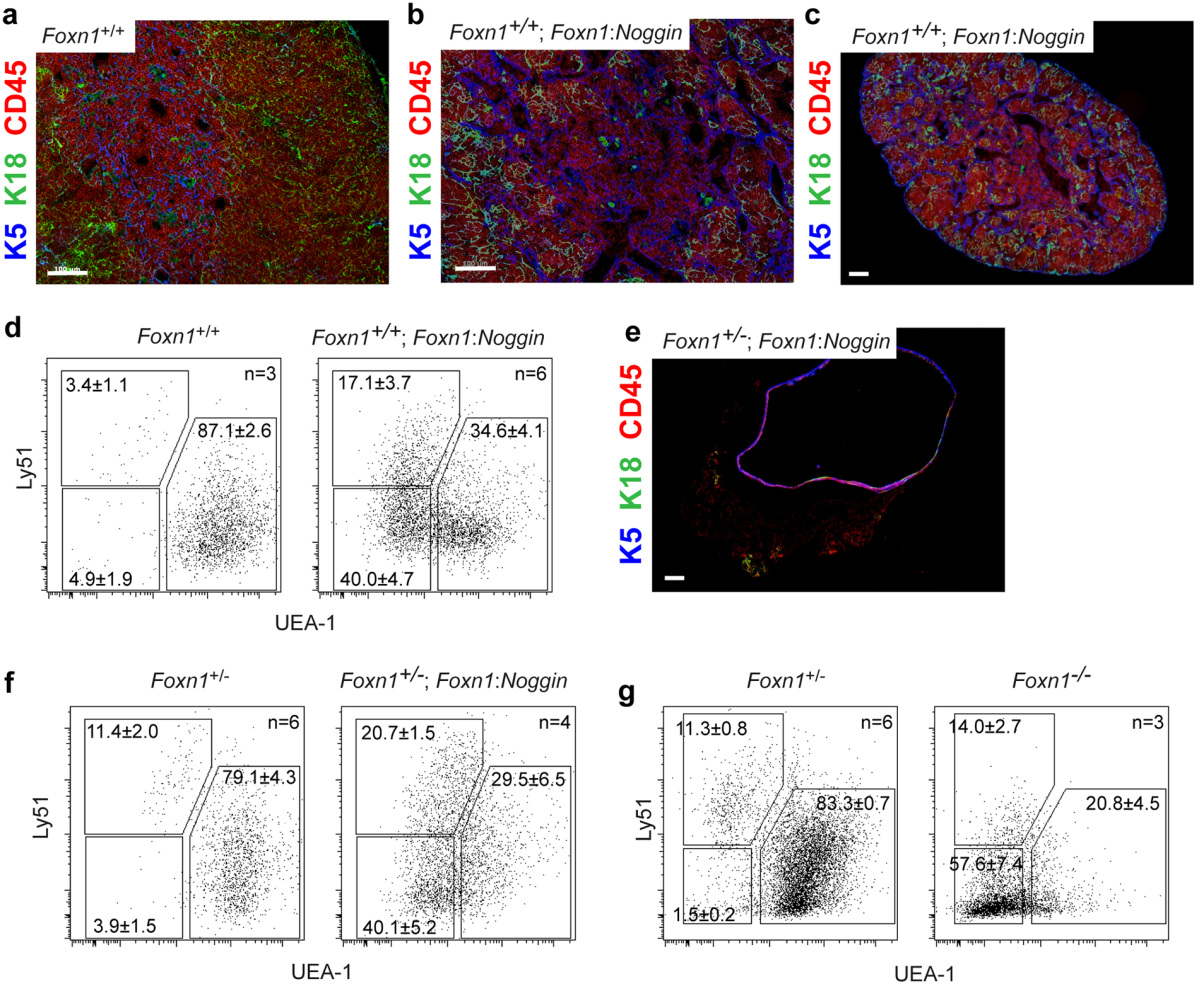
Phenotype of *Foxn1:Noggin* transgenic thymi. (**a**) Immunohistochemical appearance of wild-type thymus. The medullary epithelium is marked by expression of keratin 5 (blue fluorescence), the cortical epithelium is marked by expression of keratin 18 (green fluorescence); CD45-positive thymocytes are identified by red fluorescence. The colour code applies to all subsequent panels. (**b**) Immunohistochemistry of *Foxn1* ^+/+^;*Foxn1:Noggin* transgenic thymus. (**c**) Immunohistochemistry of *Foxn1* ^+/+^;*Foxn1:Noggin* transgenic thymus in low power view. (**d**) Flow cytometric analysis of CD45^−^EpCAM^+^ thymic epithelial cells of *Foxn1* ^+/+^;*Foxn1:Noggin* transgenic mice resolved by staining with Ly51 antibody (to mark cTEC-like cells) and UEA-1 (a marker for mTEC-like cells). Note the appearance of an aberrant population of Ly51^−/lo^/UEA-1^−^ cells. The mean percentage values (±s.e.m.) for the indicated gates are shown; for original data see Supplementary Fig. 6. (**e**) Immunohistochemistry of *Foxn1* ^+/−^;*Foxn1:Noggin* transgenic thymus. Note the cystic structure and lack of CD45-positive cells. (**f**) Flow cytometric analysis of CD45^–^EpCAM^+^ thymic epithelial cells of *Foxn1* ^+/−^;*Foxn1:Noggin* transgenic mice. The percentage values for the indicated gates are shown; for original data see Supplementary Fig. 6. (**g**) Comparative analysis of wild-type and *Foxn1* ^−/−^ thymic epithelial cells. Note the predominant Ly51^−^/UEA-1^−^ phenotype of TECs in the *Foxn1*-knock-out TECs. Mice were analysed at 4 weeks of age. Scale bars, 100 μm.

Given the low thymopoietic activity of *Foxn1:Noggin* transgenics, we next examined the structure of the microenvironment in the ectopic thymic lobes. In contrast to the clear separation of the cortical and medullary regions of a wild-type thymus, as revealed by immunohistochemistry with antibodies directed against keratin 18 and keratin 5, respectively (Fig. 3a), the small thymic lobes of *Foxn1* ^+/+^;*Foxn1:Noggin* mice exhibited an unusual structure with hypoplastic keratin 5-positive areas (Fig. 3b). The lower magnification of *Foxn1* ^+/+^;*Foxn1:Noggin* thymi shown in Fig. 3c highlights the cystic nature of the structure and an unexpected inversion of epithelial characteristics, with keratin 5-positive epithelia situated at the rim of the thymus instead of being located within the lobe. Using Ly51 and UEA-1 as markers of cTEC- and mTEC-like cells, we noted the presence of an unusual population of Ly51^−/lo^/UEA-1^−^ epithelial cells (Fig. 3d; Supplementary Fig. 6). The aberrant surface phenotype of the thymic epithelium is magnified in *Foxn1* ^+/−^;*Foxn1:Noggin* mice, in which the thymus consists of a large cyst; keratin 5-positive epithelial cells occupy a basal location and only few, if any, haematopoietic cells are present (Fig. 3e). The Ly51^−/lo^/UEA-1^−^ cell surface phenotype of transgenic epithelial cells now predominates (Fig. 3f; Supplementary Fig. 6), in line with the associated minimal thymopoietic activity; this is a clearly different situation to that in the *Foxn1*-deficient epithelium, in which the majority of cells are entirely negative for the Ly51 and UEA-1 markers (Fig. 3g).

**Figure 4:**
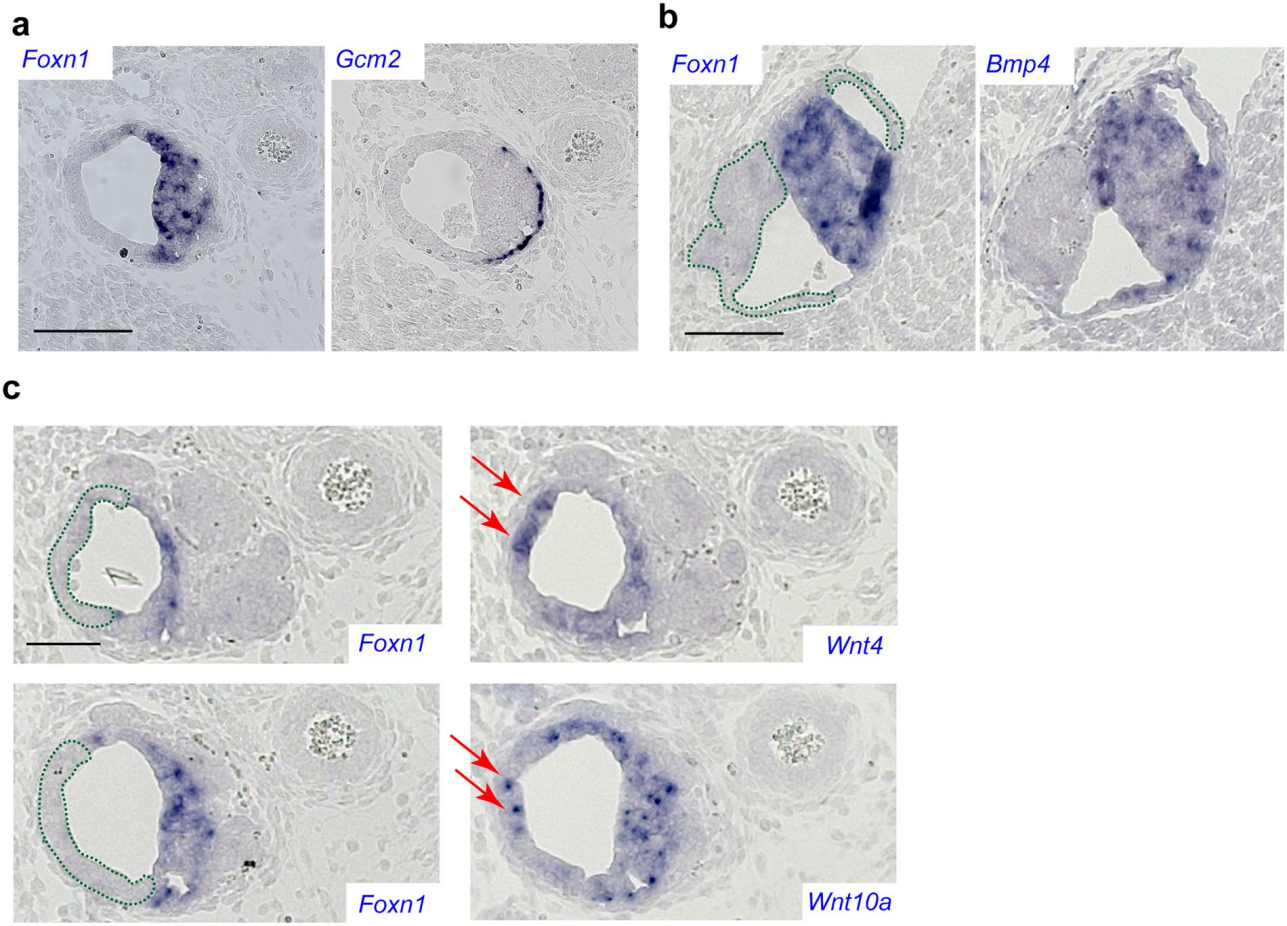
Unusual phenotype of thymic epithelia as revealed by RNA *in situ* hybridization. (**a**) Apposition of thymic (*Foxn1*-positive) and parathyroid (*Gcm2*-positive cells) in *Foxn1* ^+/+^; *Foxn1:Noggin* mice. (**b**) Lack of *Bmp4* expression in *Foxn1*-negative epithelia in *Foxn1* ^+/+^; *Foxn1:Noggin* mice; the results for two consecutive sections are shown. (**c**) Expression of *Wnt4*, and *Wnt10a* genes in the *Foxn1*-negative compartment of the thymic rudiment of *Foxn1* ^+/+^; *Foxn1:Noggin* mice. Regions of *Foxn1*-negative TECs are marked with green dotted lines in (**b**) and (**c**). Red arrows point to *Foxn1*-negative cells expressing ectopic genes in panel (**c**). Mice were analysed at day 15.5 of embryonic development. The probes used are indicated in the top left corner in panels (a) and (b), and in the bottom right corner in panel (c); positive cells are stained blue. Scale bars, 100 μm.

Next, we examined the expression profile of *Foxn1*-negative cells in the transgenic rudiment by RNA *in situ* hybridization using additional gene probes. Given the close developmental relationship of the thymic rudiment and the developing parathyroid gland, as well as the expression of *Noggin* in the parathyroid anlage (presumably to suppress BMP signalling in this region), we initially hypothesized that the emerging *Foxn1*-negative epithelium in the thymic rudiment might adopt a parathyroid fate. However, this is not the case, since *Foxn1*-negative cells do not express *Gcm2*, the defining marker of the parathyroid; instead, *Gcm2*-positive cells are found closely attached to the thymic rudiment in variable configurations (c.f., Fig. 4a; Supplementary Fig. 2a). Of note, the thymic rudiment of *Foxn1* ^−/−^ mice still expresses *Foxn1* and *Bmp4* (Supplementary Fig. 1). By contrast, the *Foxn1*-negative TECs, which emerge as a result of impaired BMP signalling, express neither (Fig. 4b), indicating that the canonical expression characteristics of typical thymic epithelia are lost. We observed that the *Foxn1*-negative cells express *Wnt4* and *Wnt10a* genes (Fig. 4c); these genes are typically expressed in mesenchymal cells of the pharyngeal region, but also in the thymic epithelium^23^, albeit independently of *Foxn1* activity (data not shown).

## Discussion

This paper addresses a potential mechanism by which reduced BMP signalling and *Foxn1* heterozygosity interact to impact the number of functional TECs. We demonstrate that impaired BMP signalling results in the loss of *Foxn1* expression in pharyngeal epithelia, and thereby decreases the number of functional TECs in the thymic rudiment. Remarkably, concomitant reduction of *Foxn1* levels drastically aggravates this phenotype.

### Transient requirement for BMP signals in early stages of thymopoiesis

Mesenchymal BMP signals initiate expression of both *Foxn1* and *Bmp4* ^6^ in endodermal cells; moreover, it is likely that activation of *Bmp4* expression in the epithelium initiates an autoregulatory positive feedback loop^24^ that maintains *Bmp4* and *Foxn1* expression in the future thymic epithelium. Since the presumptive promoter regions of *Foxn1* do not contain canonical Smad binding sites (data not shown), it appears unlikely that BMP signalling acts directly on *Foxn1* expression. Rather, BMP signalling might in parallel induce the establishment of a genetic network in thymic epithelial cells to maintain expression of the Foxn1 transcription factor. If, however, BMP signalling is extinguished early in the development of the thymic rudiment via the action of BMP inhibitors (in the present case NOGGIN), the expression of both *Foxn1* and *Bmp4* genes in the epithelium rapidly ceases. As a result, a substantial fraction of epithelial cells in the thymus exist in a non-functional, *Foxn1*-negative state. Importantly, after the BMP-sensitive phase of thymopoiesis, NOGGIN emanating from the residual *Foxn1*-expressing TECs appears to be functionally inert; this suggests that the genetic network maintaining *Foxn1* expression in TECs has by then become independent of BMP signals. This model explains previous findings that BMP signals are required for only a short period during the early phases of thymopoiesis^6^. We also demonstrate that in *Foxn1:Noggin* transgenic mice, the phenotype of epithelia after a somatically acquired state of *Foxn1*-deficiency is different from that of heritable *Foxn1*-deficiency, as indicated by discordant gene expression patterns. More specifically, genetically *Foxn1*-deficient TECs remain in a latent state for extended periods of time and can become functional stromal cells upon supply of a wild-type *Foxn1* gene^25^. By contrast, *Foxn1*-deficiency as a result of insufficient BMP signalling during the early phases of TEC development likely establishes an alternative epithelial fate unrelated to canonical thymic stroma.

Our transgenic system was designed to exhibit self-inactivating properties, in order to test whether the BMP-mediated thymic epithelial differentiation process is sensitive to developmental timing. This proved to be the case, since *Foxn1* expression cannot be re-established even after the removal of the BMP blockade. It appears therefore that sufficient BMP signals are available to the thymic rudiment only during a certain developmental window, possibly associated with a particular microenvironment. We note that normal mouse development entails drastic remodelling of spatial tissue relationships (migration of thymus to the mediastinum), which breaks down in the transgenic situation, as evidenced by the failure of the separation of parathyroid and thymic rudiments. These morphogenetic changes during development may provide an explanation for the time-sensitivity of TEC formation. This conclusion is supported by our previous observation that cervical thymi^26^ develop at a later stage than the thoracic thymus^15^, possibly owing to a different signalling environment in the neck.

### *Foxn1 gene* copy number influences the fitness of TECs

Using tetraparental chimaeras, it was previously shown that the niche maintaining thymic epithelial progenitors is of finite size; *Foxn1*-deficient thymic epithelial cells appear to be situated in the relatively quiescent progenitor niche of the thymus, thus permanently reducing the supply of thymopoietically competent TECs arising from neighbouring *Foxn1* ^+/+^ progenitors^27^. Our results are compatible with this notion, since the functional TEC compartment in *Foxn1* ^+/+^;*Foxn1:Noggin* transgenic mice does not appreciably recover postnatally.

However, our observations also indicate that *Foxn1* gene dosage affects the fitness of thymic epithelial cells. Unlike their *Foxn1* ^+/+^ counterparts, cells heterozygous for *Foxn1* are at a disadvantage when competing with the reprogrammed epithelia present in the thymus of *Foxn1:Noggin* transgenic mice. As judged from the overall number of TECs, the proliferative capacity of *Foxn1*-negative TECs and *Foxn1*-expressing TECs appears to be similar in *Foxn1* ^+/+^;*Foxn1:Noggin* transgenic mice as compared to those in their wild-type siblings. Since the thymopoietic activity of the mosaic thymus only modestly decreases from embryonic to adult stages as compared to that in the non-transgenic control, it appears that *Foxn1*-negative and *Foxn1*-positive cells proliferate at approximately equal rates. By contrast, the thymopoietic indices of *Foxn1* ^+/−^;*Foxn1:Noggin* transgenic mice are reduced by more than three orders of magnitude, despite similar overall numbers of TECs in the thymi of *Foxn1:Noggin* transgenic mice. Hence, this suggests that over time, the thymopoietically competent fraction must have become a small minority under conditions of *Foxn1* heterozygosity, when compared to the embryonic situation with a relatively large contribution of *Foxn1*-expressing cells. The ensuing cystic transformation of the postnatal transgenic thymus represents a considerable challenge for future studies addressing the spatial distribution of the remaining thymopoietically active TECs and associated developing thymocytes.

Given the qualitative changes observed for the epithelial compartment in the thymus of *Foxn1:Noggin* transgenic mice, we believe that these results cannot provide an explanation for the phenomenon of reduced output of thymocytes under conditions of *Foxn1* heterozygosity^16–20^. Indeed, the observation that the thymopoietic index of *Foxn1* ^+/−^ thymi is indistinguishable from that of *Foxn1* ^+/+^ thymi supports the conclusion that *Foxn1* haploinsufficiency is caused by quantitative, but not qualitative, changes in the TEC compartment. How *Foxn1* heterozygosity affects proliferative capacity in the TEC compartment is currently unknown, but may be related to diminished responses to proliferative signals, such as those mediated by the Fgfr2IIIb receptor expressed on thymic epithelial cells^28^.

### Epistatic control of thymopoiesis

The synthetic genetic interaction observed here between the magnitude of BMP signalling and *Foxn1* gene dosage has important implications. Gene dosage of BMP4 is known to affect developmental traits^29^, and the genetic background affects the degree of ectodermal pathology associated with *Foxn1* heterozygosity in humans^30^; it is therefore conceivable that even subtle changes in BMP signalling and Foxn1 expression levels could lead to unexpectedly severe effects with respect to thymopoietic activity. However, it is likely that factors other than BMP signalling and *Foxn1* dosage also impact on the formation of the initial TEC progenitor pool, giving rise to complex genetic interactions with unexpected outcomes; such phenomena may underlie sporadic thymic dysplasia in human patients, for instance those with Nezelof syndrome^31^. As a further illustration of this possibility, a recent study in mice has identified a quantitative trait locus on chromosome 3 near marker D3Mit127 (situated at 143 Mb) affecting the number of thymocytes and interacting with a locus on the X chromosome^32^; interestingly, the gene for BMPR1B, an essential component of the BMP receptor, is located on chromosome 3 (at 141 Mb) within the critical interval, raising the possibility that differences in the strength of BMP signalling (in this case influenced by an unknown gene on chromosome X) underlie this phenotypic variation.

## Methods

### Mice

The *Foxn1:Noggin* transgenic mice were described earlier^9^, as were *Foxn1* knock-out mice^4^. Mice were kept in the animal facility of the Max Planck Institute of Immunobiology and Epigenetics under specific pathogen-free conditions. All animal experiments were performed in accordance with the relevant guidelines and regulations, approved by the review committee of the Max Planck Institute of Immunobiology and Epigenetics and the Regierungspräsidium Freiburg, Germany (licence AZ 35-9185.81/G-12/85).

### Histology

Embryos for haematoxylin and eosin staining and RNA *in situ* hybridisation were fixed in 4% PFA and subsequently embedded in paraffin using standard techniques.

### RNA *in situ* hybridization

RNA *in situ* hybridisation (ISH) on paraffin sections was performed using DIG-labelled probes as described^33^. Sequences for the various probes used are as follows: *Foxn1*, nucleotides 2181–3584 in Genbank Accession number XM_006532266.3; *Gcm2*, nucleotides 615–1540 in Genbank Accession number NM_008104.2; *GzmA*, nucleotides 211–1019 in Genbank Accession number M13226.1; *Bmp4*, nucleotides 834–1337 in Genbank Accession number NM_001316360.1; *Wnt4*, nucleotides 340–1094 in Genbank Accession number NM_009523.2; *Wnt10a*, nucleotides 153–1407 in Genbank Accession number NM_009518.2.

### Image analysis

Images were acquired on a Zeiss Axioplan 2 microscope equipped with an AxioCam MRc 5 camera. *Foxn1*-positive and *Foxn1*-negative areas in the embryonic thymus were determined after RNA *in situ* hybridization.

### Immunofluorescence

Sections from paraformaldehyde (PFA)-fixed, paraffin-embedded tissue were de-paraffinised in xylene, re-hydrated, and then subjected to antigen retrieval with Tris-EDTA buffer (10 mM Tris, 1 mM EDTA, 0.05% Tween 20, pH 9.0) in a steamer for 17 minutes. Sections were subsequently washed in PBS-Tween 20 (0.05%) and permeabilised with 1% TritonX-100. Antibody staining was performed at room temperature in staining buffer (PBS supplemented with 4% serum and 0.05% BSA). Sections were stained for 1 hour with primary antibodies (see Supplementary Table 1), and then for 30 minutes with secondary antibodies and streptavidin. Sections were washed with PBS-Tween 20 (0.5%) between incubations. After staining, sections were mounted in Fluoromount G and images were acquired on a Zeiss Imager Z1 with ApoTome attachment using an Axiocam MRm camera.

### Flow cytometry

To generate single cell suspensions for TEC staining, thymi were finely minced with scissors, and then digested with a cocktail of collagenase type 4 (200 µg/mL), neutral protease (200 µg/mL) and DNaseI (500 ng/mL) in RPMI 1640 + 2% FCS for up to 90 minutes at 37 °C with agitation. Following digestion, EDTA was added to a final concentration of 2 mM to disaggregate any remaining cell clumps; this procedure consistently resulted in a single-cell suspension of thymocytes and stromal cells. Cells were then washed with RPMI 1640 + 2% FCS, and then re-suspended in PBS/BSA for staining. Thymocyte suspensions for CD4/CD8 staining were generated by gently pressing thymic lobes through 40 µm sieves. Cell surface staining (see Supplementary Table 1 for antibodies) was performed at 4 °C in PBS supplemented with 0.5% BSA and 0.02% NaN_3_. Thymic epithelial cells have the surface phenotype CD45^−^/EpCAM^+^; thymocytes are CD45^+^/EpCAM^−^.

### Statistical analysis

t-tests (two-tailed) were used to determine the significance levels of the differences between the means of two independent samples, considering equal or unequal variances as determined by the F-test. For multiple tests, the conservative Bonferroni correction was applied.

## Electronic supplementary material

Supplementary Information
