# Supplementary material for: Cooperative interaction of BMP signalling and Foxn1 gene dosage determines the size of the functionally active thymic epithelial compartment
Source: Sci Rep. 2017 Aug 17;7:8492. doi: 10.1038/s41598-017-09213-1 (PMC5561201; doi:10.1038/s41598-017-09213-1)
Supplement: Supplementary file 1 — Supplementary Information [file 41598_2017_9213_MOESM1_ESM.pdf]

Supplementary information for

Cooperative interaction of BMP signalling and *Foxn1* dosage determines the size of the functionally active thymic epithelial compartment

Jeremy B. Swann, Brigitte Krauth, Christiane Happe, Thomas Boehm

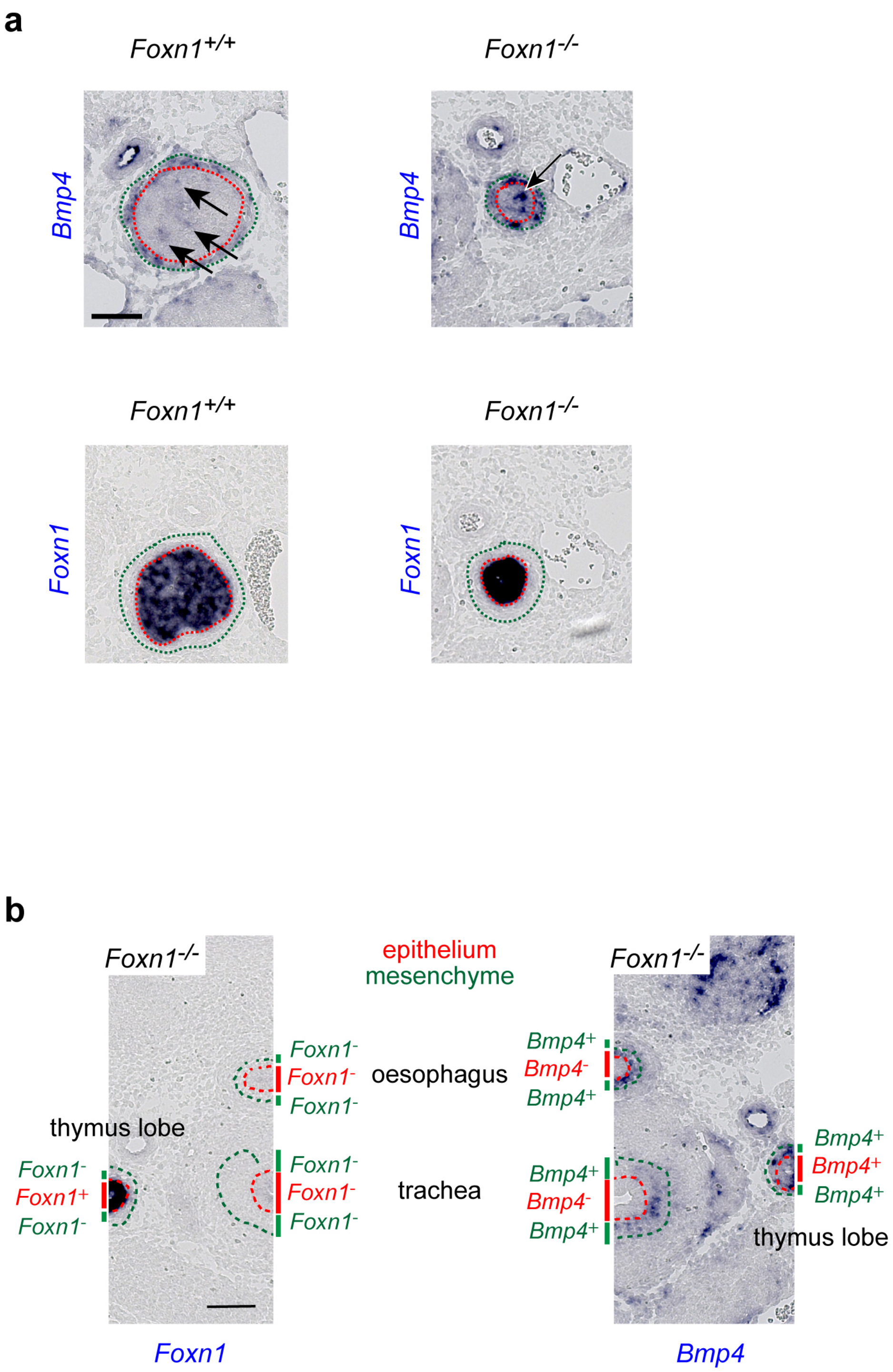
a
Foxn1+/+
Foxn1-/-
Bmp4
Bmp4
Foxn1+/+
Foxn1-/-
Foxn1
Foxn1
b
Foxn1-/-
epithelium
mesenchyme
Foxn1-/-
Foxn1-
Foxn1-
Foxn1-
oesophagus
Bmp4+
Bmp4-
Bmp4+
thymus lobe
Foxn1-
Foxn1+
Foxn1-
Foxn1-
Foxn1-
Foxn1-
trachea
Bmp4+
Bmp4-
Bmp4+
Bmp4+
Bmp4+
Bmp4+
thymus lobe
Foxn1
Bmp4

Supplementary Figure 1. Expression of *Bmp4* and *Foxn1* genes in the thymic anlage. (**a**) Expression of *Bmp4* and *Foxn1* genes in the thymus of E13.5 wild-type (*Foxn1*$^{+/+}$) (left panels) and Foxn1-knock out (*Foxn1*$^{-/-}$) mice as revealed by RNA *in situ* hybridization (positive cells are stained blue); the epithelial compartments are encircled with dotted red lines, the borders of organotypic mesenchymal capsules with green lines. Note that the *Foxn1*-probe used here detects transcripts from both wildtype and mutant *Foxn1* alleles. In contrast to somatically reprogrammed epithelia (see **Fig. 4b**), cells in *Foxn1*$^{-/-}$ thymic rudiments continue to express *Foxn1* and *Bmp4* transcripts. (**b**) Expression profiles of *Foxn1* and *Bmp4* genes in pharyngeal organs of *Foxn1*$^{-/-}$ mice at E13.5. The epithelial and mesenchymal compartments of thymus, oesophagus and trachea are highlighted in colour code and the presence of detectable expression of *Foxn1* and *Bmp4* indicated by "+" or "-" signs. Note that in contrast to the thymic epithelium, epithelia in the oesophagus and trachea do not express *Bmp4*.

Scale bars, 100 μm.

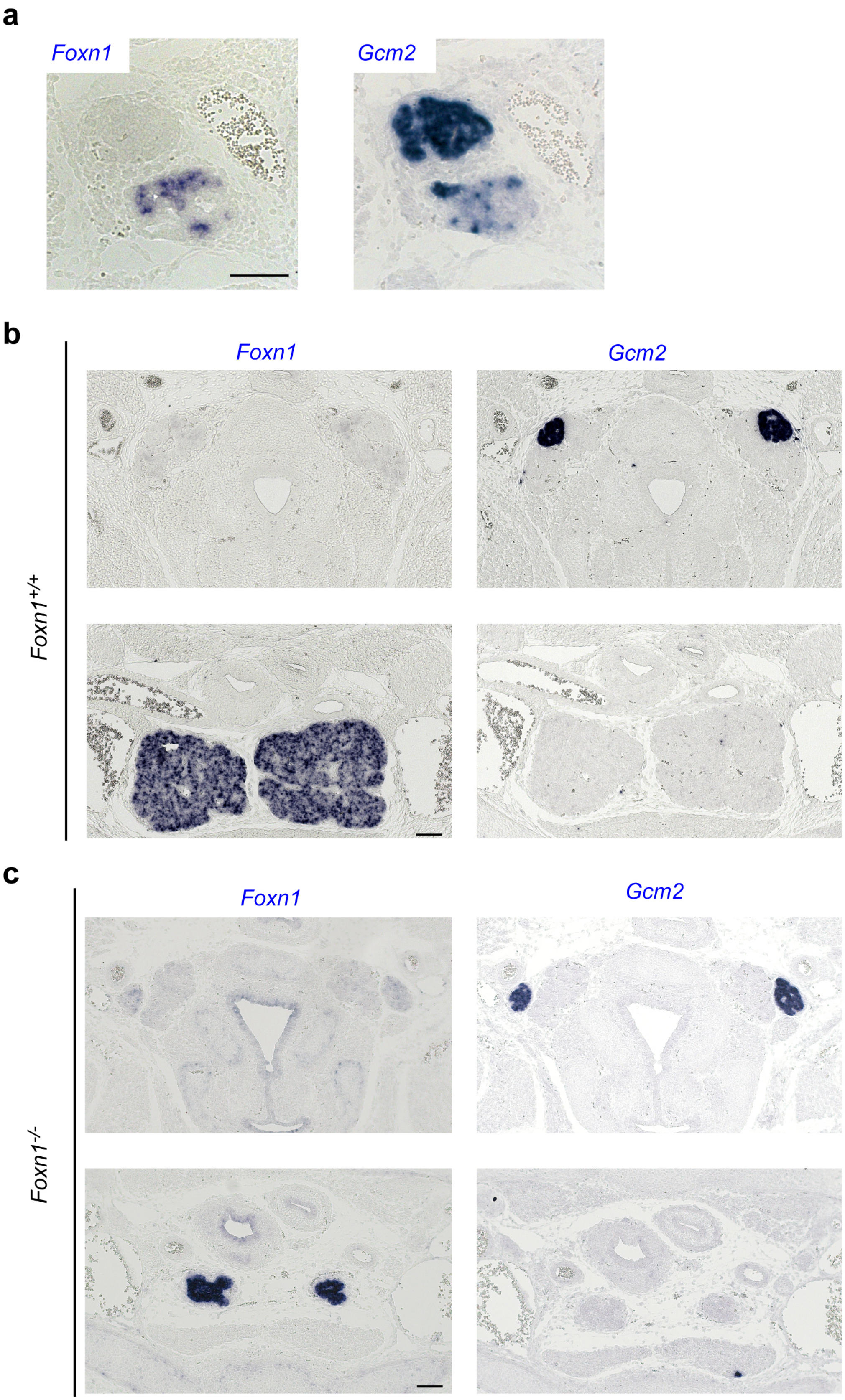
a
Foxn1
Gcm2
b
Foxn1
Gcm2
Foxn1+/+
c
Foxn1
Gcm2
Foxn1-/-

Supplementary Figure 2. Spatial relationship of thymus and parathyroid in wild-type and transgenic mice.

(**a**) Failure of separation of thymus and parathyroid anlagen in *Foxn1*$^{+/+}$; *Foxn1:Noggin* mice as revealed by *in situ* hybridization with thymus (*Foxn1*) and parathyroid (*Gcm2*) specific probes. Note that in *Foxn1:Noggin*-transgenic embryos the combined thymus/parathyroid anlagen are located ectopically, anterior to the thyroid. For comparison, the normal separation of the thymus and parathyroid anlagen in wild-type (**b**) and *Foxn1*$^{-/-}$ **(c)** embryos is depicted. Separation of the thymus (*Foxn1*-positive cells in the mediastinum, left panels in **(b)** and **(c)**) and parathyroid (*Gcm2*-positive cells embedded in thyroid lobes, right panels in **(b)** and **(c)**) is evident for both genotypes. Mice were analysed at day 15.5 of embryonic development; positive cells are stained blue. Scale bars, 100 μm.

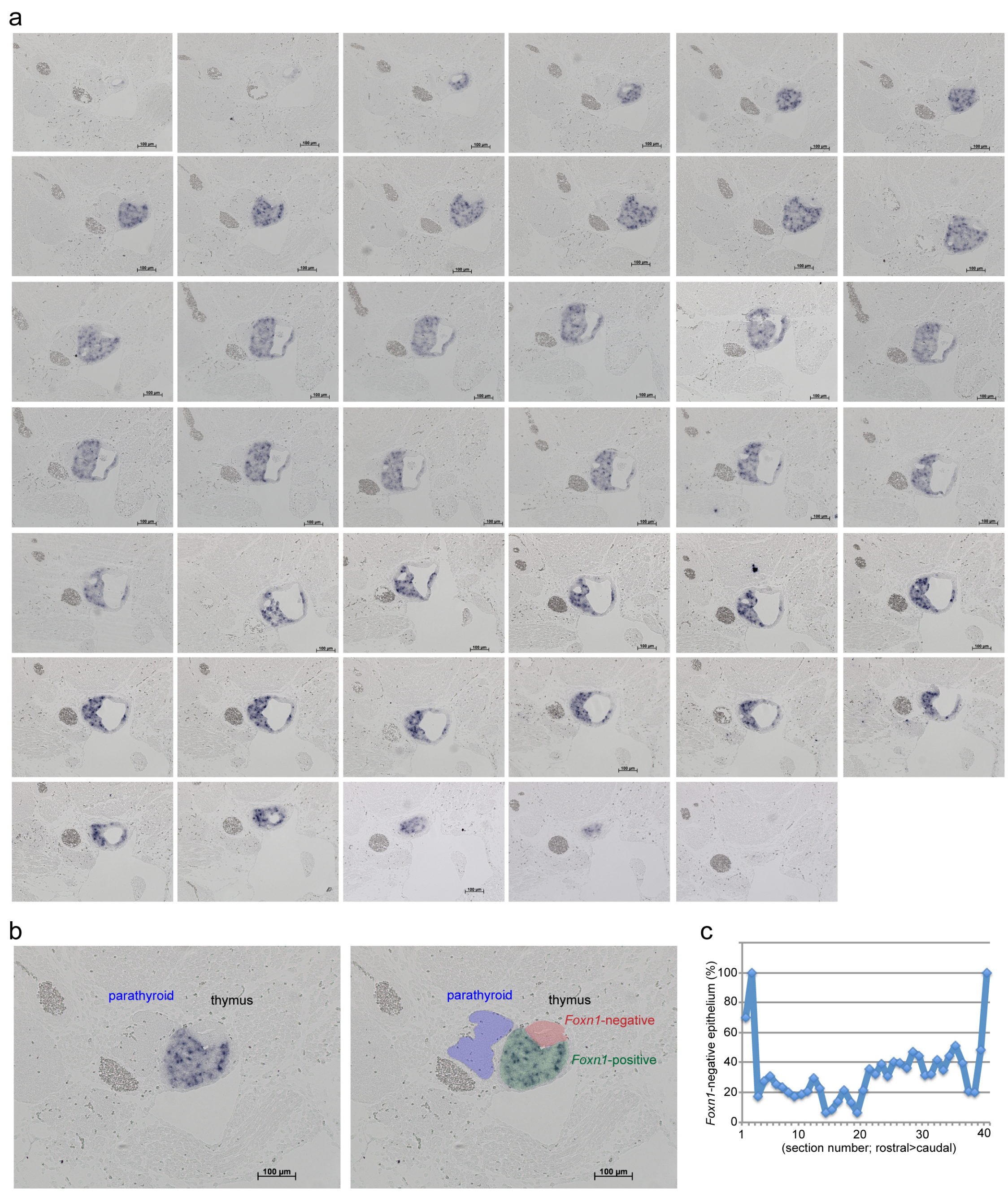

Supplementary Figure 3. Thymopoietic activity in the thymic rudiments of *Foxn1*$^{+/+}$;*Foxn1:Noggin* transgenic mice.

(**a**) RNA *in situ* hybridization of serial sections (6µm) of a thymic rudiment from a *Foxn1*$^{+/+}$; *Foxn1:Noggin* embryo at day 15.5 of embryonic development. *Foxn1*-positive cells are stained blue. Sections are arranged serially from left to right, top to bottom;

section #1 is in the top left corner. (**b**) Representative section from the series to indicate the mosaic pattern of Foxn1 expression in the rudiment. The shaded regions in the right panel indicate the *Foxn1*-positive (green shading) and *Foxn1*-negative (red shading) areas used to calculate the proportions of *Foxn1*-negative epithelium depicted in **(c)**. **(c)** Fraction of *Foxn1*-negative epithelium as a function of anatomical position. These values are summarily depicted in **Fig. 1g**. Sections 1 and 41 represent the cranial and rostral poles of the thymic rudiment respectively.

Representative data of two biological replicas. Scale bars, 100 μm.

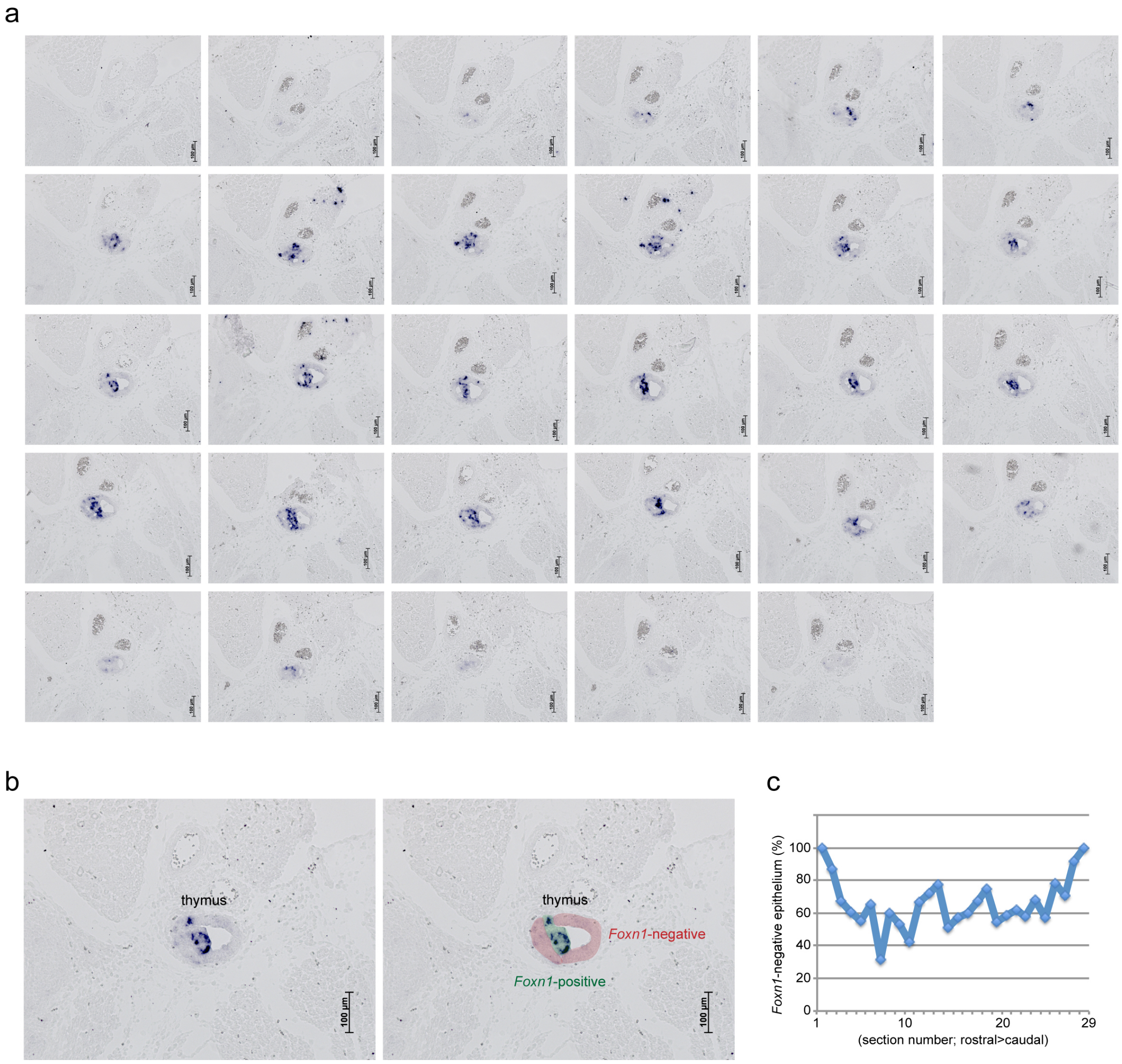

Supplementary Figure 4. Thymopoietic activity in the thymic rudiments of *Foxn1*$^{+/-}$;*Foxn1:Noggin* transgenic mice.

RNA *in situ* hybridization of serial sections (6µm) of a thymic rudiment from a *Foxn1*$^{+/-}$; *Foxn1:Noggin* embryo at day 15.5 of embryonic development. *Foxn1*-positive cells are stained blue. (**b**) Representative section from the series to indicate the mosaic pattern of Foxn1 expression in the rudiment. The shaded regions in the right panel indicate the *Foxn1*-positive (green shading) and *Foxn1*-negative (red shading) areas used to calculate the proportions of *Foxn1*-negative epithelium depicted in **(c)**. **(c)** Fraction of *Foxn1*-

negative epithelium as a function of anatomical position. These values are summarily depicted in **Fig. 1g**. Sections 1 and 29 represent the cranial and rostral poles of the thymic rudiment respectively.

Representative data of two biological replicas. Scale bars, 100 μm.

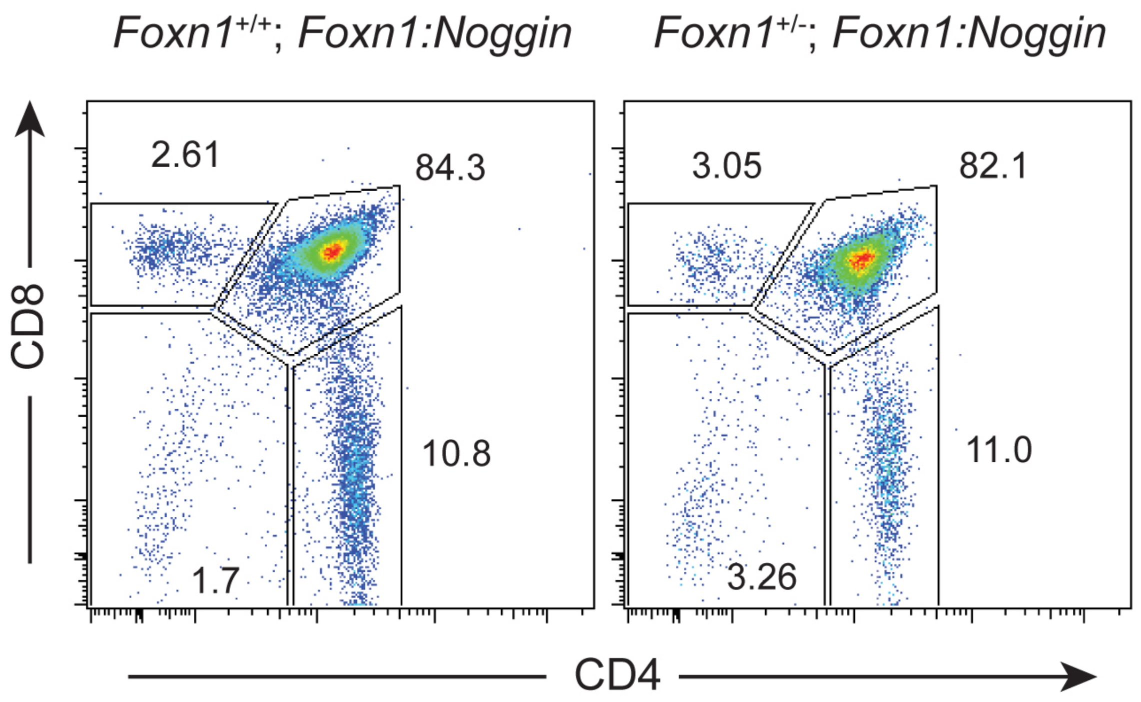

Supplementary Figure 5. Normal T cell development in the thymic rudiments of *Foxn1:Noggin* transgenic mice.

Indicated are representative flow cytometric results obtained by CD4 and CD8 staining of thymocytes isolated from the ectopic thymi of the indicated genotypes. These patterns do not differ from those of wild-type and *Foxn1* heterozygous mice.

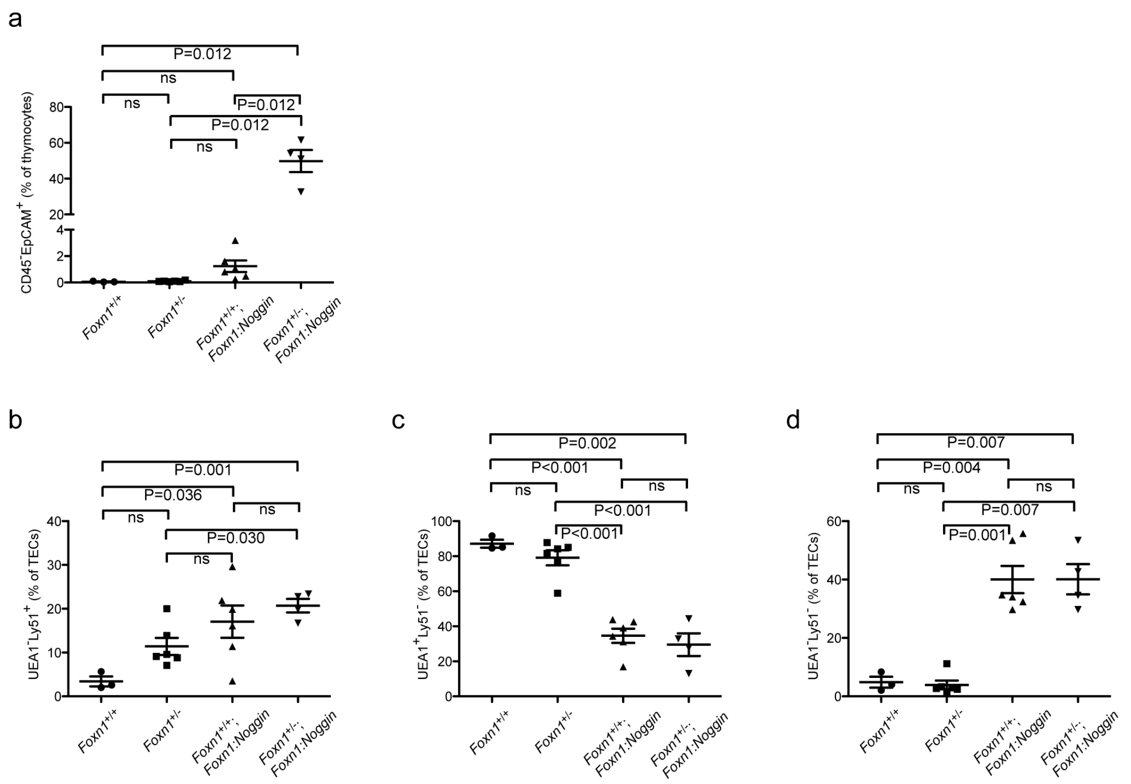

Supplementary Figure 6. Quantification of thymic epithelial cells according to their surface phenotype.

(**a**) Fraction of TECs in total thymocytes. (**b**) Fraction of TECs with cortical phenotype. (**c**) Fraction of TECs with medullary phenotype. (**d**) Fraction of TECs with undetermined ("double-negative") phenotype.

Mice of the indicated genotypes were analysed at 4 weeks of age. Means±s.e.m. are shown.

Supplementary Table 1. Antibodies and staining reagents for flow cytometry and immunofluorescence

**Antigen/Reagent**	**Clone**	**Conjugate**	**Supplier**
CD3	145-2C11	APC	eBioscience
CD4	GK1.5	FITC	BioLegend
CD8	53-6.7	PE	eBioscience
CD19	1D3	PE Cy7	eBioscience
CD44	1M7	PE	BD Bioscience
CD45	30-F11	PE Cy7	BioLegend
CD62L	MEL-14	FITC	eBioscience
CD80	16-10A1	Biotin	BioLegend
EpCAM	G8.8	APC	BioLegend
Keratin 5	rabbit polyclonal	-	Covance
Keratin 8	Troma-1	-	in house
Ly51	6C3	PE	eBioscience
MHC2	M5/114.15.2	FITC	BioLegend
mouse IgG1	goat polyclonal	Biotin	SouthernBiotech
mouse IgG (H+L)	rat polyclonal	FITC	Jackson ImmunoResearch
rabbit IgG (H+L)	goat polyclonal	Alexa Fluor 488	Invitrogen
rat IgG (H+L)	donkey polyclonal	Cy3	Jackson ImmunoResearch
Streptavidin	-	Cy3, Cy5	Jackson ImmunoResearch
Streptavidin	-	FITC, PE or Alexa Fluor v450	eBioscience
UEA-1	-	Biotin, FITC	Vector Laboratories
